# β-Lactamase production and antibiotic susceptibility pattern of *Moraxella catarrhalis* isolates collected from two county hospitals in China

**DOI:** 10.1186/s12866-018-1217-5

**Published:** 2018-07-20

**Authors:** Wei Shi, Denian Wen, Changhui Chen, Lin Yuan, Wei Gao, Ping Tang, Xiaoping Cheng, Kaihu Yao

**Affiliations:** 1 Key Laboratory of Major Diseases in Children, National Key Discipline of Pediatrics (Capital Medical University), National Clinical Research Center for Respiratory Diseases, Beijing Key Laboratory of Pediatric Respiratory Infection Diseases, Beijing Pediatric Research Institute, Beijing Children’s Hospital, Capital Medical University, No. 56 Nan-li-shi Road, Beijing, 100045 China; 2 People’s Hospital of Zhongjiang County, No. 96 Da-bei Street, Zhongjiang, 618100 Sichuan China; 3 Youyang Hospital, the First Affiliated Hospital of Chongqing Medical University, People’s Hospital of Chongqing Youyang County, No. 102 Tao-hua-yuan Road, Chongqing, 409899 Youyang China

**Keywords:** *Moraxella catarrhalis*, Antibiotic resistance, β-Lactamase, Children

## Abstract

**Background:**

*Moraxella catarrhalis* (*M. catarrhalis*) is an important bacterial pathogen. However, its antibiotic susceptibility patterns in different areas are difficult to compare because of the use of different methods and judgement criteria. This study aimed to determine antimicrobial susceptibility and β-lactamase activity characteristics of *M. catarrhalis* isolates collected from two county hospitals in China, and to express the results with reference to three commonly used judgement criteria.

**Results:**

Nasopharyngeal swabs were obtained from child inpatients with respiratory tract infections at the People’s Hospital of Zhongjiang County and Youyang County from January to December 2015. *M. catarrhalis* strains were isolated and identified from the swabs, and susceptibility against 11 antimicrobials was determined using the E-test method or disc diffusion. Test results were interpreted with reference to the standards of the European Committee on Antimicrobial Susceptibility Testing (EUCAST), the Clinical and Laboratory Standards Institute (CLSI), and the British Society for Antimicrobial Chemotherapy (BSAC). Detection of β-lactamase activity was determined by the chromogenic cephalosporin nitrocefin. *M. catarrhalis* yield rates were 7.12 and 9.58% (Zhongjiang County, 77/1082 cases; Youyang County, 101/1054 cases, respectively). All isolates were susceptible to amoxicillin–clavulanic acid. The susceptibility rate to meropenem was 100% according to EUCAST; no breakpoints were listed in CLSI or BSAC. The non-susceptibility rate to sulfamethoxazole-trimethoprim differed significantly between the two hospitals regardless of the judgemnet criteria used, with isolates from Zhongjiang showing higher susceptibility to those from Youyang (Fisher’s exact test, *P* < 0.05). According to CLSI, the total non-susceptibility rate to erythromycin was 70.8% (Zhongjiang County, 79.2%; Youyang County, 64.3%), and the rate reached 92.1% (Zhongjiang County, 90.9%; Youyang County, 93.1%) on the basis of EUCAST or BSAC. The total positive rate of β-lactamase was 99.4% (177/178 cases) (Zhongjiang County, 100%, 77/77 cases; Youyang County, 99.0%, 100/101 cases).

**Conclusions:**

Ninety nine percent of *M. catarrhalis* isolates produce β-lactamase. The isolates showed poor susceptibility to ampicillin and erythromycin, and high susceptibility to the third- and fourth-generation cephalosporins and amoxicillin–clavulanic. Significant discrepancies between different antimicrobial susceptibility judgemnet criteria were noted.

## Background

*Moraxella catarrhalis* (*M. catarrhalis*), also known as *Micrococcus catarrhalis*, *Neisseria catarrhalis*, and *Branhamella catarrhalis* is a gram-negative, aerobic, oxidase-positive diplococcus. It is a commensal species that is exclusively a human pathogen of the upper respiratory tract. However, it is now recognized as causative of otitis media in children and acute exacerbations of chronic obstructive pulmonary disease in adults [1, 2]. *M. catarrhalis* can also cause endocarditis, sepsis and meningitis, though these occur more rarely [1].

Community-acquired pneumonia (CAP) is a major cause of morbidity and mortality in children worldwide, and *M. catarrhalis* is the third most common causative pathogenic bacteria of CAP, following *Streptococcus pneumoniae* and *Haemophilus influenzae* [3]. The importance of *M. catarrhalis* in CAP infection is also increasing with the introduction of the pneumococcal conjugate vaccine and *Haemophilus influenzae* type b vaccine.

The first β-lactamase positive *M. catarrhalis* isolate was reported in 1977 in Sweden [4]. Since then, β-lactamase production has been reported from various countries with different frequencies, which mostly exceed 90.0% [3, 5–7], revealing the poor antibiotic susceptibility pattern of the isolate. Three antimicrobial resistance judgemnet criteria can be used to analyze the antibiotic susceptibility pattern of *M. catarrhalis* strains: the European Committee on Antimicrobial Susceptibility Testing (EUCAST), the Clinical and Laboratory Standards Institute (CLSI) [8], and the British Society for Antimicrobial Chemotherapy (BSAC) [9]; CLSI is most commonly used in China. Because of the lack of a unified testing method and judgemnet criteria for antibiotic resistance of *M. catarrhalis*, data from different areas show significant differences [10, 11]. Moreover, susceptibility testing of *M. catarrhalis* is not routinely performed in most diagnostic laboratories, and many resistance data derive from large cities, with few studies conducted in more rural areas. Therefore, the aim of this study was to determine the level of β-lactamase production and antibiotic resistance patterns of *M. catarrhalis* isolates collected from two county hospitals in China.

## Methods

### Study population

Nasopharyngeal swabs were collected from child inpatients with respiratory tract infections at the People’s Hospitals of Zhongjiang County and Youyang County, located in western China, from January to December 2015. Children meeting the following criteria were included: (1) aged between 1 month and 14 years; (2) having an infection for fewer than 3 days; (3) having a fever and cough; and (4) with permission from a parent and/or legal guardian. Children with factors potentially affecting the acquisition of nasopharyngeal swab specimens, such as repeated tic, and coagulation disorders, were excluded.

A total of 1082 children were included from Zhongjiang County, of whom 642 were male and 440 were female; a total of 1054 were included from Youyang County, of whom 679 were male and 375 were female. Chocolate agar and blood agar were used to culture the specimens, which were incubated for 24 h at 37 °C. Bacterial growth was identified by the examination of colony morphology, gram staining, and microscopic analysis. Additional identification tests were the oxidase test, catalase test, carbohydrate fermentation test, DNAse test, and nitrate reduction test.

A parent and/or legal guardian of each participant signed a written informed consent document before enrollment and before any study procedure was performed. This study was approved by the Ethics Committee of the two hospitals (Ethics Committee of People’s Hospital of Zhongjiang County, and Ethics Committee of People’s Hospital of Chongqing Youyang County). No ethical problems were encountered in this study.

### Antimicrobial susceptibility and β-lactamase testing

The minimum inhibitory concentrations (MICs) of all isolates were determined against ampicillin, amoxicillin–clavulanic acid, cefuroxime, ceftazidime, cefepime, ciprofloxacin, erythromycin, and meropenem using E-test strips (AB Biodisk, Solna, Sweden), and their susceptibility to sulfamethoxazole–trimethoprim, chloramphenicol, and tetracycline was assessed with disc diffusion tests (Oxoid, Basingstoke, England). The results were interpreted with reference to the standards of EUCAST(http://www.eucast.org/clinical_breakpoints/), CLSI [8], and BSAC [9], and breakpoints are listed in Table 1. β-lactamase activity was determined by the chromogenic cephalosporin nitrocefin (BR66A; Oxoid). *Streptococcus pneumoniae* ATCC49619 was used as the quality control strain in susceptibility tests.

**Table 1:** Antibiotic breakpoints for M. catarrhalis strains in EUCAST, CLSI and BSAC

Antibiotics^*^	MIC^a^/D-zone^b^	EUCAST^c^		CLSI^d^			BSAC^e^		
		S	R	S	I	R	S	I	R
AMP	MIC(mg/L)	-^#^	-^#^	-^#^	-^#^	-^#^	≤1	–	> 1
AMC	MIC(mg/L)	≤1	>1	≤4	–	≥8	≤1	–	> 1
CXM	MIC(mg/L)	≤4	>8	≤4	8	≥16	≤1	2	> 2
CAZ	MIC(mg/L)	-^#^	-^#^	≤2	-^#^	-^#^	-^#^	-^#^	-^#^
FEP	MIC(mg/L)	≤4	>4	-^#^	-^#^	-^#^	-^#^	-^#^	-^#^
CIP	MIC(mg/L)	≤0.5	>0.5	≤1	-^#^	-^#^	≤0.5	–	> 0.5
ERY	MIC(mg/L)	≤0.25	>0.5	≤0.5	1–4	≥8	≤0.25	0.5	> 0.5
MEM	MIC(mg/L)	≤2	>2	-^#^	-^#^	-^#^	-^#^	-^#^	-^#^
TCY	D-zone(mm)	≥28	<25	≥29	25–28	≤24	≥22	–	≤21
CHL	D-zone(mm)	≥30	<30	-^#^	-^#^	-^#^	-^#^	-^#^	-^#^
SXT	D-zone(mm)	≥18	<15	≥13	11–12	≤10	≥12	–	≤11

^a^*MIC* minimum inhibitory concentration, ^b^*D-zone* diameter of inhibition zone, ^c^*EUCAST*, European Committee on Antimicrobial Susceptibility Testing, ^d^*CLSI* Clinical and Laboratory Standards Institute, ^e^*BSAC* British Society for Antimicrobial Chemotherapy, ^*^AMP ampicillin, *AMC* amoxicillin–clavulanic acid, *CXM* cefuroxime, *CAZ* ceftazidime, *FEP* cefepime, *CIP* ciprofloxacin, *ERY* eryciprofloxacin, *MEM* meropenem, *TCY* tetracycline, *CHL* chloramphenicol, *SXT* sulfamethoxazole–trimethoprim, ^#^ no breakpoints listed in the according criterion

### Statistical analysis

Antimicrobial resistance data were analyzed with WHONET 5.6 software as recommended by the World Health Organization. Statistical analyses were performed using SPSS software 17.0 (SPSS, Chicago, IL). The χ^2^ test and Fisher’s exact test were used for significant comparisons between groups. *P* values < 0.05 were deemed to indicate statistical significance.

## Results

A total of 77 and 101 *M. catarrhalis* isolates were separately detected from Zhongjiang and Youyang counties, respectively, with yield rates of 7.12% (77/1082 cases) and 9.58% (101/1054 cases). There was no significant difference in the yield rate between boys (6.7%; 43/642 cases) and girls (7.7%; 34/440 cases) in Zhongjiang County (*χ*^2^ = 0.419, *P* = 0.518), or in Youyang County (9.0%; 61/679 cases and 10.7%; 40/375 cases, respectively, χ^2^ = 0.790, *P* = 0.374).

Table 2 shows the susceptibility and the MICs of the isolates against 11 antibiotics. All isolates were susceptible to amoxicillin–clavulanic acid. The susceptibility rate to meropenem was 100% according to EUCAST; no equivalent breakpoints are listed in CLSI or BSAC. Regardless of the criteria used, the strains showed poor susceptibility to erythromycin. According to CLSI, the total non-susceptibility rate to erythromycin was 70.8% (Zhongjiang County, 79.2%; Youyang County, 64.3%), and this reached 92.1% (Zhongjiang County, 90.9%; Youyang County, 93.1%) according to EUCAST or BSAC. The MICs of 21.9% (39/178 cases) of the isolates were > 256 mg/L. Non-susceptibility to sulfamethoxazole-trimethoprim was significantly different between the two hospitals, regardless of the judgemnet criteria used, with isolates from Zhongjiang more susceptible than those from Youyang (Fisher’s exact test, *P* < 0.05). The total positive rate of β-lactamase was 99.4% (177/178 cases): 99.0% (100/101 cases) in Youyang County and 100% (77/77 cases) in Zhongjiang County.

**Table 2:** Susceptibility of the Moraxella catarrhalis isolates in Youyang, Zhongjiang and total profiles according to EUCAST, CLSI and BSAC

	Antibiotics^*^	Youyang					Zhongjiang					Total				
		I%	R%	MIC50	MIC90	MIC Range	I%	R%	MIC50	MIC90	MIC Range	I%	R%	MIC50	MIC90	MIC Range
EUCAST^a^	AMP	–	–	2	4	0.016~ 16	–	–	2	6	0.032~ 12	–	–	2	6	0.032–12
	AMC	–	0	0.19	0.38	0.016~ 0.38	–	0	0.25	0.25	0.016~ 0.38	–	0	0.25	0.25	0.016–0.38
	CXM	8.9	0	2	4	0.023~ 8	18.2	1.3	3	6	0.047~ 16	12.9	0.6	3	6	0.047–16
	CAZ	–	–	0.125	0.38	0.023~ 2	–	–	0.094	0.38	0.032~ 1	–	–	0.094	0.38	0.032–1
	FEP	–	4.0	1.5	4	0.064~ 6	–	1.3	1.5	4	0.19~ 6	–	2.8	1.5	4	0.19–6
	CIP	–	2.0	0.047	0.19	0.016~ 1	–	5.2	0.047	0.094	0.016~ 1	–	3.4	0.047	0.094	0.016–1
	ERY	28.7	64.4	0.75	> 256	0.047~ > 256	11.7	79.2	2	> 256	0.19~ > 256	21.3	70.8	2	256	0.19- > 256
	MEM	–	0	0.006	0.008	0.003~ 0.38	–	0	0.006	0.008	0.003~ 0.023	–	0	0.006	0.008	0.003–0.38
	TCY	3.0	5.9				0	3.9				1.7	5.1			
	CHL	–	4.0				–	3.9				–	3.9			
	SXT	9.9	30.7				7.8	3.9				9.0	19.1			
CLSI^b^	AMC	–	0	0.19	0.38	0.016~ 0.38	–	0	0.25	0.25	0.016~ 0.38	–	0	0.25	0.25	0.016–0.38
	CXM	8.9	0	2	4	0.023~ 8	18.2	1.3	3	6	0.047~ 16	12.9	0.6	3	6	0.047–16
	CAZ	-^#^	-^#^	0.125	0.38	0.023~ 2	-^#^	-^#^	0.094	0.38	0.032~ 1	-^#^	-^#^	0.094	0.38	0.032–1
	CIP	-^#^	-^#^	0.047	0.19	0.016~ 1	-^#^	-^#^	0.047	0.094	0.016~ 1	-^#^	-^#^	0.047	0.094	0.016–1
	ERY	48.5	15.8	0.75	> 256	0.047~ > 256	44.1	35.1	2	> 256	0.19~ > 256	47.8	23.0	2	> 256	0.19- > 256
	TCY	20.8	5.9				3.9	3.9				13.5	5.1			
	SXT	6.9	16.8				0	1.3				3.9	10.1			
BSAC^c^	AMP	–	68.3	2	4	0.016~ 16	–	81.8	2	6	0.032~ 12	–	74.2	2	6	0.032–12
	CXM	45.5	49.5	2	4	0.023~ 8	44.2	53.2	3	6	0.047~ 16	44.9	51.1	3	6	0.047–16
	TCY	–	5.9				–	3.9				–	5.1			
	SXT	–	19.8				–	1.3				–	11.8			

^a^*EUCAST* European Committee on Antimicrobial Susceptibility Testing, ^b^*CLSI* Clinical and Laboratory Standards Institute, ^c^*BSAC* British Society for Antimicrobial Chemotherapy, ^*^AMP ampicillin, *AMC* amoxicillin–clavulanic acid, *CXM* cefuroxime, *CAZ* ceftazidime, *FEP* cefepime, *CIP* ciprofloxacin, *ERY* eryciprofloxacin, *MEM* meropenem, *TCY* tetracycline, *CHL* chloramphenicol, *SXT* sulfamethoxazole–trimethoprim, ^#^ no breakpoints were listed in the according criterion, but the other “-“s mean no data

## Discussion

Research into the antibiotic resistance of *M. catarrhalis* strains is limited, and the use of different judgemnet criteria makes it difficult to compare data between studies.Three criteria are commonly used in antimicrobial susceptibility testing for *M. catarrhalis*: EUCAST, CLSI, and BSAC. Clinical studies in China most commonly use CLSI. All isolates in the present study were susceptible to amoxicillin–clavulanic, ceftazidime, and ciprofloxacin according to CLSI, indicating that these antibiotics could be used for empirical treatment. The non-susceptibility rate to cefuroxime showed a significant difference according to different judgement criteria, being 13.5% according to CLSI and EUCAST, but 96.0% according to BSAC. This is a source of confusion for clinicians. Previous reports documented high susceptibility rates of *M. catarrhalis* isolates to third- and fourth-generation cephalosporins (such as ceftriaxone and ceftazidime) and amoxicillin–clavulanic, at nearly 100% [9, 11–14].

Isolates in the present study showed high resistance to erythromycin. Previous studies reported that 23S rRNA mutations A2330T and A2058T are major causes of resistance to macrolide antibiotics such as erythromycin [15–17]. Because the resistance of pathogens isolated from the respiratory tract to macrolide antibiotics is a serious concern in China, it is important to standardize the use of this antibiotic to help control and reduce the spread of resistance.

The β-lactamase positive rate in our study was high, at 99.4% (177/178 cases), which is in accordance with other recent findings (96.5%~ 100%) in China [14, 18, 19]. According to EUCAST, *M. catarrhalis* isolates producing β-lactamase should be reported as resistant to penicillins and aminopenicillins without inhibitors. However, we showed a resistance rate to ampicillin of 74.2% (Zhongjiang County, 81.8%; Youyang County, 68.3%) according to BSAC, suggesting that there is a discrepancy between β-lactamase production and resistance rates. We advise medical workers to pay attention to the high resistance rate and possible discrepancies in rates during their routine work.

## Conclusions

In summary, 99% of *M. catarrhalis* strains of the present study were shown to produce β-lactamase. The isolates showed high susceptibility to third- and fourth-generation cephalosporins and amoxicillin–clavulanic, and poor susceptibility to ampicillin and erythromycin. However, some notable discrepancies were observed between susceptibility rates obtained with different antibiotic resistance judgemnet criteria for *M. catarrhalis* isolates. Therefore, additional long-term surveys are required to monitor the antimicrobial resistance of this important human pathogen with the aim of standardizing criterion.

## Abbreviations

BSAC: British Society for Antimicrobial Chemotherapy
CAP: Community-acquired pneumonia
CLSI: Clinical and Laboratory Standards Institute
EUCAST: European Committee on Antimicrobial Susceptibility Testing
MIC: Minimum inhibitory concentration
